# Supplementary material for: Exploratory Study of the Relationship between an Oral Fungal Swab Test and Patient Blood Test Data
Source: Microorganisms. 2023 Nov 29;11(12):2887. doi: 10.3390/microorganisms11122887 (PMC10745972; doi:10.3390/microorganisms11122887)
Supplement: Supplementary file 1 [file microorganisms-11-02887-s001.zip › microorganisms-2733211-supplementary.pdf]

Table S1. Dataset on preliminary investigation.

age	*Candida*	sex
39	0	Female
70	1	Female
76	0	Female
70	1	Male
50	1	Female
72	1	Female
53	0	Female
61	0	Male
80	1	Female
74	0	Female
81	1	Female
78	1	Female
48	0	Female
73	1	Female
57	1	Female
81	0	Female
69	0	Female
66	0	Male
77	1	Female
54	0	Female
72	1	Female
64	1	Female
73	1	Male
43	0	Male
75	1	Female
44	0	Female
83	1	Female
88	1	Male
66	0	Male
79	0	Female
79	1	Female
88	0	Female
60	0	Female
49	0	Female
71	1	Male
64	1	Female

87	0	Female
62	1	Female
84	0	Female
86	1	Male
90	1	Female
73	0	Female
51	0	Female
48	1	Female
67	0	Female
63	0	Female
66	1	Female
75	1	Male
91	1	Female
80	1	Female
51	0	Female
46	0	Female
86	1	Male
50	1	Female
72	1	Female
79	1	Female
80	0	Female
75	1	Female
76	0	Male
67	1	Male
44	0	Male
77	1	Female
62	0	Male
77	1	Female
67	0	Female
35	0	Female
76	0	Male
79	1	Female
71	1	Female
38	0	Female
62	1	Male
41	0	Female
41	1	Female
94	1	Male

83	0	Female
41	0	Female
73	1	Female
61	0	Female
67	0	Female
84	1	Male
67	0	Male
76	0	Male
69	0	Female
59	0	Female
75	0	Female
68	0	Female
71	0	Female
56	1	Female
77	0	Male
67	0	Female
68	0	Female
53	0	Female
75	1	Female
69	0	Female
72	1	Female
61	0	Male
47	0	Male
59	0	Female
62	0	Female
74	1	Female
56	1	Female
55	1	Female
81	1	Female
50	0	Female
53	0	Male
74	0	Male
60	0	Male
54	1	Female
67	0	Female
68	0	Female
71	0	Female
49	0	Female

58	1	Female
64	0	Female
75	1	Male
57	0	Male
56	0	Female
81	1	Female
73	0	Male
79	0	Female
52	0	Female
84	0	Female
59	0	Female
66	1	Male
78	0	Male
78	0	Female
55	0	Male
46	0	Female
78	1	Female
72	1	Female
72	0	Male
50	1	Female
51	0	Female
54	0	Female
65	0	Female
75	0	Male
72	0	Female
55	0	Female
73	1	Female
36	1	Male
76	1	Male
52	0	Female
78	0	Female
74	0	Female
77	0	Female
48	0	Female
71	0	Male
76	1	Female
41	0	Female
67	0	Female

65	0	Female
79	1	Female
75	1	Female
73	0	Female
71	0	Female
64	0	Female
57	0	Female
64	0	Male
75	0	Female
68	1	Female
71	0	Female
71	0	Female
63	0	Female
56	0	Female
77	0	Female
43	0	Female
58	0	Female
49	1	Female
67	1	Female
58	1	Male
52	0	Male
58	0	Female
56	1	Female
20	0	Male
59	0	Male
47	0	Female
59	0	Female
58	0	Female
57	0	Female
71	0	Male
72	0	Female
67	0	Female
62	0	Female
78	0	Female
65	0	Female
72	0	Male
63	0	Female
48	1	Female

50	0	Female
59	1	Female
45	0	Female
57	1	Female
76	0	Female
65	0	Male
66	0	Female
66	0	Male
52	0	Female
51	1	Male
69	1	Female
65	1	Male
55	0	Male
43	1	Male
75	0	Male
65	0	Female
78	0	Female
47	0	Female
47	1	Female
71	1	Female
83	0	Female
66	0	Male
66	0	Male
75	0	Male
80	1	Female
71	1	Male
66	0	Female
90	0	Female
44	1	Female
48	0	Female
67	0	Female
84	0	Female
54	0	Female
77	0	Female
76	1	Female
75	0	Female
74	1	Female
72	0	Female

75	0	Female
69	0	Female
58	0	Female
50	0	Female
59	0	Female
56	0	Female
73	1	Male
62	0	Female
75	1	Female
19	0	Female
76	0	Male
86	0	Female
63	0	Female
71	0	Male
66	1	Female
75	0	Female
67	1	Female
72	0	Male
62	1	Male
48	0	Female
69	1	Female
73	0	Male
72	0	Female
44	0	Male
63	0	Female
66	0	Male
52	0	Female
68	0	Male
67	0	Male
69	0	Male
26	0	Female
79	1	Female
80	1	Female
72	1	Female
73	1	Male
74	1	Female
89	1	Male
53	0	Female

73	1	Male
73	0	Female
76	1	Male
77	1	Female
81	1	Female
69	0	Female
64	1	Female
68	1	Female
76	0	Female
54	1	Female
71	0	Female
75	1	Female
64	0	Female
71	0	Female
72	0	Female
65	0	Female
74	0	Male
77	0	Female
75	0	Male
50	0	Female
68	0	Female
79	1	Male
73	0	Female
71	0	Female
67	0	Female
81	1	Female
66	0	Female
57	0	Female
57	0	Female
72	1	Male
63	1	Female
59	0	Female
47	0	Male
19	1	Male
56	1	Male
52	0	Female
66	0	Female
79	0	Female

56	1	Female
64	1	Female
33	0	Female
69	1	Female
68	1	Female
56	0	Male
66	0	Female
82	1	Male
24	1	Female
63	0	Female
73	1	Female
73	1	Male
74	1	Male
62	1	Female
56	0	Female
84	0	Female
86	1	Female
75	0	Female
65	0	Female
68	0	Male
74	0	Female
74	1	Female
67	0	Female
81	0	Female
82	0	Male
61	0	Male
57	0	Female
72	1	Male
79	1	Female
57	0	Female
70	1	Female
52	0	Female
63	1	Female
68	0	Male
82	0	Male
76	0	Female
54	1	Female
71	0	Female

75	1	Female
72	0	Female
77	1	Male
55	0	Female
72	1	Female
37	0	Female
83	1	Female
70	0	Female
74	0	Female
85	1	Female
65	0	Male
73	1	Female
64	0	Female
67	1	Female
77	0	Female
47	0	Female
67	1	Female
36	0	Female
62	0	Female
83	0	Female
55	0	Female
79	1	Female
80	1	Male
69	0	Male
71	1	Male
76	1	Female
58	1	Female
62	0	Female
79	0	Female
49	1	Female
60	0	Female
65	0	Female
68	1	Male
82	0	Male
67	0	Female
53	0	Female
63	1	Female
71	1	Female

62	1	Female
80	1	Female
51	1	Female
80	1	Male
65	0	Female
76	1	Female
56	0	Female
81	1	Male
71	1	Female
54	1	Female
86	1	Female
68	1	Female
85	1	Female
74	1	Female
43	0	Female
73	0	Male
73	1	Female
88	1	Male
56	1	Female
55	1	Female
51	0	Female
50	0	Female
77	1	Female
68	1	Female
54	1	Female
62	1	Female
62	0	Female
68	0	Female
71	0	Female
66	0	Male
72	0	Male
66	1	Male
54	0	Male
68	1	Female
57	0	Male
71	1	Female
64	0	Female
75	1	Male

57	0	Male
71	0	Female
81	1	Female
73	0	Male
79	0	Female
46	0	Female
53	0	Female
51	0	Female
